# Latency and Amplitude of Cortical Activation in Interactive vs. Passive Tasks: An fNIRS Study Using the NefroBall System

**DOI:** 10.3390/s25134135

**Published:** 2025-07-02

**Authors:** Karolina Jezierska, Agnieszka Turoń-Skrzypińska, Iwona Rotter, Anna Syroka, Magdalena Łukowiak, Kamila Rawojć, Piotr Rawojć, Aleksandra Rył

**Affiliations:** 1 Department of Medical Physics, Pomeranian Medical University, ul. Ku Słońcu 13, 71-073 Szczecin, Poland; magdalena.lukowiak@pum.edu.pl (M.Ł.); piotr.rawojc@pum.edu.pl (P.R.); 2 Department of Medical Rehabilitation and Clinical Physiotherapy, Pomeranian Medical University in Szczecin, ul. Żołnierska 54, 71-210 Szczecin, Poland; agneszka.turon.skrzypinska@pum.edu.pl (A.T.-S.); iwona.rotter@pum.edu.pl (I.R.); anna.syroka@pum.edu.pl (A.S.); aleksandra.ryl@pum.edu.pl (A.R.); 3 Maria Skłodowska-Curie National Institute of Oncology, ul. Garncarska 11, 31-115 Cracow, Poland; kamila.rawojc@dworska.pl

**Keywords:** functional near-infrared spectroscopy, cortex activation, rehabilitation therapies, Nefroball

## Abstract

Functional near-infrared spectroscopy (fNIRS) allows non-invasive assessment of cortical activity during naturalistic tasks. This study aimed to compare cortical activation dynamics—specifically the latency (t_max_) and amplitude (ΔoxyHb) of oxygenated haemoglobin changes—in passive observation and an interactive task using the Nefroball system. A total of 117 healthy adults performed two tasks involving rhythmic hand movements: a passive protocol and an interactive game-controlled condition. fNIRS recorded signals from the visual, parietal, motor, and prefrontal cortices of the left hemisphere. The Mann–Whitney test revealed significantly shorter t_max_ in all areas during the interactive task, suggesting faster recruitment of cortical networks. ΔoxyHb amplitude was significantly higher only in the visual cortex during the interactive task, indicating increased visual processing demand. No significant ΔoxyHb differences were observed in the motor, prefrontal, or parietal cortices. Weak but significant positive correlations were found between tmax and ΔoxyHb in the motor and prefrontal regions, but only in the passive condition. These findings support the notion that interactive tasks elicit faster, though not necessarily stronger, cortical responses. The results have potential implications for designing rehabilitation protocols and brain–computer interfaces involving visual–motor integration.

## 1. Introduction

Research on brain activity in motor and cognitive tasks is an important area of modern human sciences, combining issues from neurology, physiology, and biomedical engineering. In recent years, non-invasive functional brain imaging methods have gained popularity, allowing for monitoring of neuronal processes in conditions close to natural ones. One such method is functional Near-Infrared Spectroscopy (fNIRS) [1]. Thanks to the simplicity of measurement, relatively small restrictions on the movement of participants, and low load on the subjects, fNIRS facilitates experiments in various cognitive and motor contexts [1,2,3].

fNIRS measures changes in the cerebral cortex’s oxygenated (HbO) concentration and reduced haemoglobin (HbR) concentration. fNIRS instruments use infrared light, which penetrates the soft tissues of the skull and is partially absorbed by the blood. Differences in the degree of radiation absorption are related to the level of haemoglobin oxygenation, allowing us to conclude that there is an increase in oxygen demand in active brain areas. An increase in HbO concentration compared to HbR concentration indicates local neuronal activity [1,4].

Numerous experiments have demonstrated the usefulness of fNIRS in analysing motor, premotor, or prefrontal cortex activation during motor tasks. For example, researchers have used it to assess the intensity of motor cortex engagement during precise hand movements or stroke rehabilitation exercises. At the same time, this method allows for the recording of activity in other areas responsible for processing visual information, sensory integration, or attention control, which expands the possibilities of interpretation and enables a comprehensive understanding of the processes [3,5,6].

fNIRS gains particular importance in the context of games and interactive rehabilitation exercises. It provides insight into the dynamics of brain activation during tasks in which the patient or healthy participant takes real actions (e.g., controlling a virtual object) while simultaneously monitoring the effects on an ongoing basis. Thanks to this, it is possible to assess the level of involvement of different brain regions and examine their activation rate, i.e., the so-called latency time [6,7].

A common practice in designing experiments with fNIRS is comparing different tasks. The brain’s response to passive tasks (e.g., simple schematic movements) is often compared to active tasks involving planning and movement in response to stimuli. This allows us to see the influence of engagement and attention. The theory of motor learning assumes that active tasks induce more complex recruitment of neuronal networks, primarily when the participant must simultaneously assess the situation and the correct movement to achieve the intended goal (e.g., hitting a virtual object) [8]. The results of many studies indicate that in interactive tasks, the motor and prefrontal cortex are more strongly activated than in situations of passively viewing the stimulus, which translates into greater blood flow in these areas. Importantly, however, some studies report a similar level of maximum activation but significant differences in the dynamics of the response, especially in the time after which the peak value of haemoglobin oxygenation appears. A shorter delay (latency) may suggest faster engagement of cognitive–motor resources in an interactive task than in a passive one [9,10].

Nefroball is one example of an active rehabilitation or training system in which the control of a virtual object (an aeroplane) is coupled with the performance of specific movements or physical exercises (e.g., pressing a ball). The device used in this study is a custom-made prototype constructed at the Pomeranian Medical University (PUM), Szczecin, Poland, and is not commercially available (protected by a Polish patent application (Application No. P.444325) and granted exclusive rights (Patent No. PAT.247408, granted on 9 April 2025). Systems like this stimulate the muscular system and require visual engagement, motor–spatial coordination, and rapid response. Consequently, we can expect increased and faster activation of the visual cortex and areas responsible for movement planning and control, compared to conditions where the participant only passively observes the stimulus. The primary motivation for conducting such research is to understand better how interactive tasks affect brain activity compared to passive tasks. Precise data on the intensity and dynamics of activation can indicate effective strategies for engaging different brain areas. To date, there has been a lack of detailed studies focusing on the dynamics of cortical activation, including fNIRS signal latency and amplitude, in the context of interactive rehabilitation tasks. This is particularly relevant in physiotherapy, given the potential impact of patient motivation and engagement on treatment effectiveness.

This study aimed to compare the dynamics of cortical activation, particularly the latency and amplitude of HbO changes, in passive and interactive (Nefroball) conditions using fNIRS. The results of this work have potential applications in rehabilitation planning. They may be exciting in the context of therapy for patients requiring eye–hand coordination training. Furthermore, a complete understanding of the phenomenon may contribute to the optimization of educational or rehabilitation games and applications. We hypothesised that interactive tasks would lead to shorter latencies and potentially larger amplitudes in relevant cortical areas due to increased cognitive and motor engagement.

## 2. Materials and Methods

The study involved 117 individuals (78 women and 39 men) aged 19 to 41 years (median = 19 years, Q1 = 19 years, Q3 = 20). Among the participants, 16 were left-handed. Before the study began, each participant completed a general health questionnaire that included questions about chronic diseases (including neurological, motor, and ophthalmological conditions), medications taken, and overall well-being. None of the participants reported any ailments that could affect motor or cognitive functions. All subjects were neurologically healthy and did not report any ailments that could affect concentration or motor skills, including epilepsy, brain injuries, and severe vision disorders. Before starting the experiment, each participant was informed about the purpose and course of the study and gave written informed consent to participate. The local bioethics committee approved the project.

**Figure 1:**
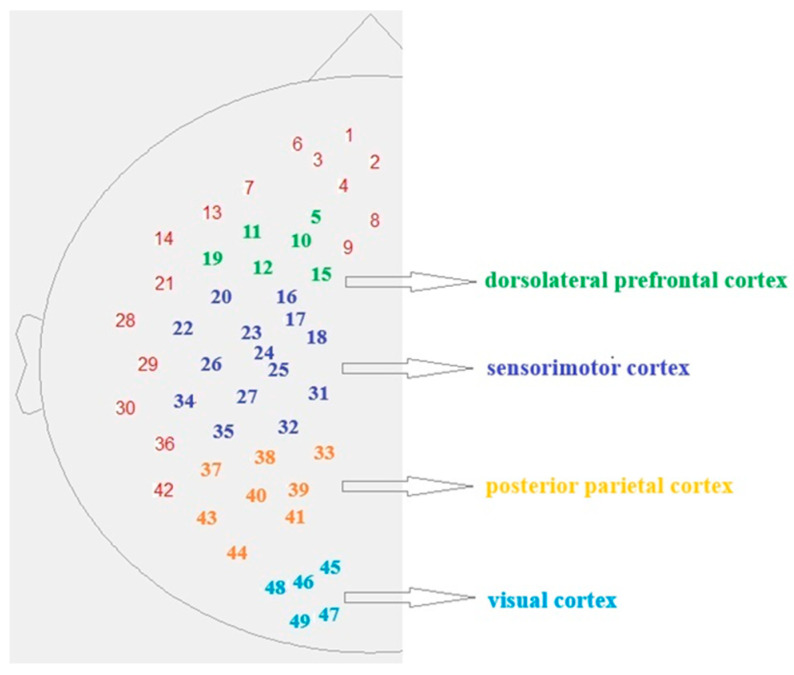
Diagram of channel arrangement on the head surface, divided into analysed areas of the cerebral cortex.

The fNIRS system (NIRScout, NIRx Medical Technologies LLC, Glean Head, NY, USA) measured brain activity with 16 near-infrared light sources and 16 detectors. The configuration of the measuring probes was designed to record signals from selected areas of the brain’s left hemisphere, including the visual (occipital), parietal, motor, and prefrontal cortex. The arrangement of the measuring channels was determined based on EEG 10–20 standards (Figure 1). The entire apparatus was placed in a room with controlled lighting conditions to limit the influence of external light.

Each participant wore an fNIRS cap with installed light sources and detectors. Before the recording began, the device was calibrated, and the signal level and fit of the probes to the head surface were checked. The participant sat in a comfortable chair about 60–70 cm from the monitor.

**Figure 2:**
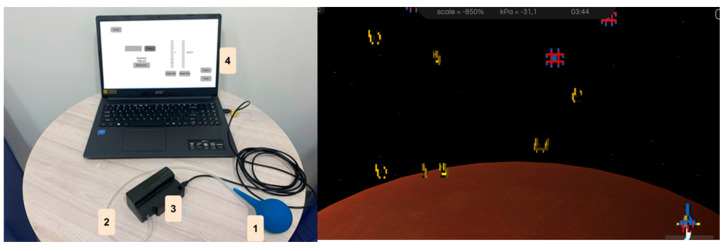
On the left—NefroBall system diagram.

The NefroBall system was designed for vascular access training (arteriovenous fistula) in patients undergoing haemodialysis. Its primary goal is to enable regular fistula training, with physicians or physiotherapists able to monitor progress remotely. Figure 2 shows the system diagram.

1. Pressure Controller: A pneumatic, spherical rubber device (SBR, NR, or silicone) with a 1 mm opening and a 2 mm PE connector. Its modular design allows different ball sizes to match the patient’s hand. It ensures airtightness and easy disinfection.

2. Connection Tube: A sealed conduit linking the controller to the measurement system, transmitting pressure changes caused by squeezing the ball.

3. Measurement System: Composed of an Atmega32U4 microcontroller and MPX5700AP pressure sensor in a compact housing. It measures force and duration of pressure, converting pressure changes into voltage and then into normalised values (0–1024). Data are sampled at 25 Hz and sent via USB to a computer.

4. Computer Application: Handles data visualisation and storage. It includes three modules:

o Patient database management;
o Standard training;
o Gamified training (e.g., Space Invaders game).

On the right is a screenshot from the game in the “Space Invaders” application.

Participants performed two tasks in a random order (protocol P and A) to minimise the effects of learning or fatigue on performing a specific task variant. The order of task performance (interactive vs. passive protocol) was alternating, with participants being assigned to either the interactive or passive task group. Each task lasted approximately 3 min, with a short rest break (1–2 min) between conditions.

Protocol P—Passive task (passive observation)—the participant was asked to rhythmically squeeze a pressure controller (approximately every 2 s) while observing a computer screen displaying an image of an aeroplane moving in space—the “Space Invaders” game (without affecting the controls). The goal was to maintain relatively uniform ball compressions without reacting to changes on the screen.

**Figure 3:**
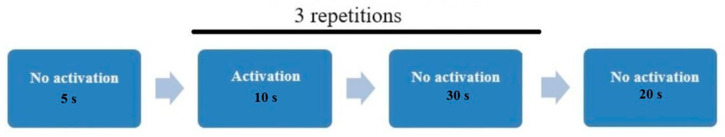
Scheme of study. Activation is stimulation with passive observation (protocol P) or an interactive task with Nefroball (protocol A). Each condition consisted of a short baseline rest, followed by three repetitions of alternating “no activation” and “activation” blocks, and a final rest. “No activation” blocks correspond to resting periods used to reduce carryover effects and allow the signal to return to baseline between tasks.

Protocol A—Interactive task (NefroBall—“Space Invaders” game)—the participant performed the same ball-squeezing movement. However, this time, each movement was recorded by the NefroBall system, allowing for shooting down flying objects. The participant’s task was to shoot down as many of the objects that appeared as possible within a specified time. The appropriate ball compression directly translated into the number of projectiles fired by the plane, which required eye–hand coordination. The same study design was used for both protocols A and P, as presented in Figure 3.

Great care was taken to ensure that the patient did not perform unnecessary activities or movements during the examination to limit possible artefacts in the optical signal. Additionally, the cap was attached directly to the chest tape with straps to limit the influence of jaw movements related to swallowing saliva.

The recording parameters were identical for both protocols. All data were analysed in NIRSLab 15.0. The recordings were band-pass filtered with a high cutoff frequency of 0.2 Hz and a low cutoff frequency of 0.01 Hz to remove slow drifts and high-frequency noise. No additional motion–artefact correction techniques were applied. However, channels with visibly abnormal signal patterns were manually inspected and excluded from analysis. Participants were instructed to minimise movements, and the optodes were secured with additional chest straps to reduce motion-related artefacts. The typical spectrum for oxyHb and deoxy-Hb used in the analyses was created based on the manufacturer’s recommendations. The final data for each participant was the average of three repetitions. Channels in which the signal was exceptionally noisy (e.g., caused by improper optode adhesion) were excluded from the analysis. Hence, the amount of analysed data differs from the number of participants examined and differs for different areas. As a result, the number of participants included in the final analysis varied by cortical region. Usable data were obtained from 97 participants in the left hemisphere (~17% excluded), 99 in the motor cortex (~15% excluded), 97 in the prefrontal cortex (~17% excluded), 88 in parietal cortex (~25% excluded), and 84 in the visual cortex (~28% excluded).

In each cerebral cortex area analysed, the maximum and minimum relative changes in oxygenated haemoglobin (HbO) were determined. The average amplitude of the increase in oxygenated oxyhaemoglobin, ΔoxyHb [mmol/L] (max–min), was calculated for each participant. For each channel, ΔoxyHb was defined as the difference between the minimum value observed in the pre-activation period and the maximum value reached during the task. This method reflects the within-trial dynamic amplitude of the haemodynamic response. No fixed baseline correction was applied. Deoxyhaemoglobin and total haemoglobin signals were not included in the current analysis. Additionally, the latency time tmax [s] was measured, i.e., the time from the beginning of the task to the HbO signal reaching its maximum value. This parameter allowed us to assess the speed of recruitment of neuronal processes in a given area.

The distribution of the obtained results was checked using the Shapiro–Wilk test. Since the data distribution for all analysed areas deviated from the normal, the nonparametric Mann–Whitney test was used to compare the results obtained in the passive and interactive conditions (Nefroball). The Spearman test was used to examine the correlation between delta and tmax. The level of statistical significance was assumed to be *p* = 0.05 (Dell Inc. 2016, Dell Statistica, data analysis software system, version 13. soft-ware.dell.com).

## 3. Results

**Figure 4:**
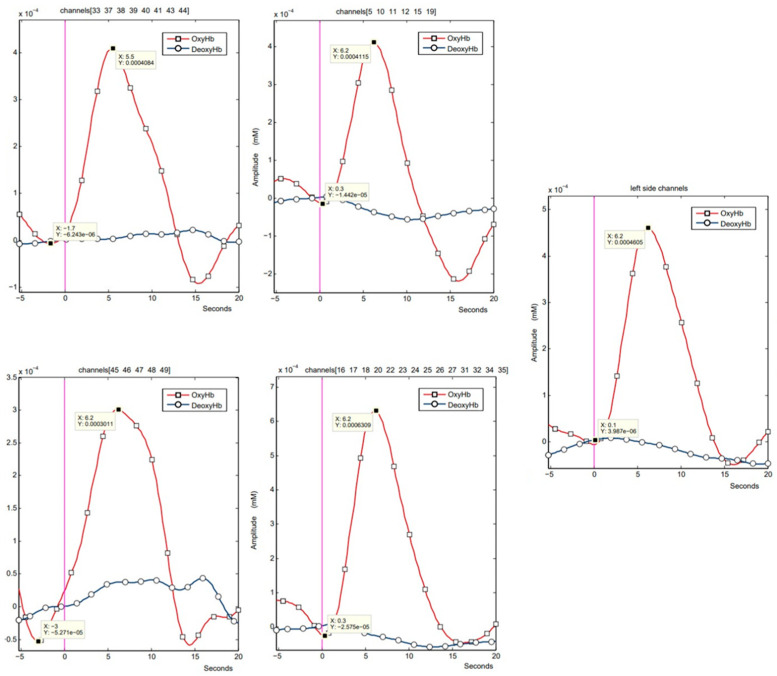
Examples of fNIRS signal recordings for the individual areas analysed. Time is shown in seconds on the x-axis; ΔHbO values are in mmol/L on the y-axis. The channel numbers listed above each subplot indicate which source–detector pairs were assigned to each cortical region.

The distribution of data for each variable was assessed using the Shapiro–Wilk test. As shown in Table 1, all variables significantly deviated from normality (*p* < 0,05), justifying the use of nonparametric statistical methods in further analyses. The data were non-normally distributed. Table 2 and Table 3 compare the median and first and third quarter values for ΔoxyHb and tmax for protocols A and P, respectively. The obtained results indicate that the onset of haemodynamic response occurred significantly earlier in the interactive condition. In terms of amplitude (ΔoxyHb), statistically significant differences were found only in the visual cortex between the conditions. Figure 4 shows examples of fNIRS signal recordings for the individual areas analysed.

Statistical analysis of fNIRS data using the Mann–Whitney test showed significant differences in tmax latencies between the interactive (Nefroball—protocol A) and passive (observation—protocol P) conditions for all analysed areas. At the same time, a statistically significant difference in ΔoxyHb was obtained only in the visual cortex. Table 4 contains information on the *p*-coefficient for individual analyses. A significant correlation was found between ΔoxyHb and tmax in the passive condition in the motor and prefrontal cortex.

**Figure 5:**
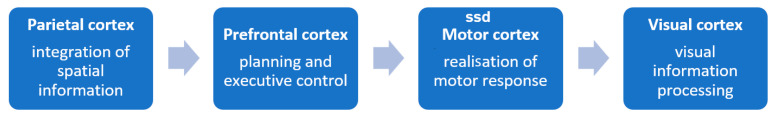
Possible direction of excitation transfer, for both protocols. The figure also identifies specific brain areas’ likely actions while performing the task.

Figure 5 illustrates the possible direction of excitation transfer, which is the same for protocols P and A, with longer latencies for the active task in each case.

Table 5 presents information on the results of the correlation analysis between ΔoxyHb and tmax. A weak, positive, statistically significant correlation was found only for the motor and prefrontal cortex for both protocols.

## 4. Discussion

The experiment compared cortical activity during passive ball squeezing combined with observation of a virtual aeroplane and in an interactive condition (Nefroball), in which hand movement translated into controlling an object in the game. Evidence shows that when we initiate movement, we engage areas of the prefrontal, premotor, motor, and parietal cortex and subcortical structures such as the cerebellum, thalamus, and basal ganglia. Similar results were reported [11,12,13]. The parietal cortex acts as an intermediary between sensory and motor representations, integrating, among other things, somatosensory and visual stimuli. This information is then passed on to the premotor cortex and primary motor cortex, where it is transformed into movement planning and execution, which is consistent with the published results of other researchers [12,14,15].

Analysis of fNIRS data showed significant differences in the dynamics of neuronal activation between the interactive (Nefroball) and passive (observation) protocols. In particular, differences were observed in the latency time, reaching the maximum value of the oxygenated haemoglobin signal (tmax), with relatively stable amplitude values (ΔoxyHb), which is consistent with the published results of other researchers [16,17,18]. As a result of the analysis of the neuronal recruitment time data for the interactive protocol (Nefroball), a significantly shorter latency time was revealed in all analysed areas of the left hemisphere; these results suggest that a task requiring integration of visual information and movement control elicits faster activation of the cortical structures responsible for planning, attention, and motor responses. Shortenings of tmax in the prefrontal and parietal cortex seem particularly significant, indicating their key role in organising goal-directed behaviour as reported in previous studies [9,10].

In the case of the ΔoxyHb signal amplitude, the differences between the interactive and passive conditions did not reach statistical significance, except for the visual cortex (*p* = 0.031), where a higher increase in HbO was noted in the Nefroball task. It can be assumed that the interactive condition is associated with a greater demand for visual information processing, which leads to a stronger activation of this area. The visual cortex was an apparent exception to the above pattern; here, the interactive task generated a higher signal amplitude. This can be interpreted as a greater demand on the visual processing resources associated with tracking objects and making quick decisions about shooting them down. In other words, controlling a virtual aircraft naturally increases the perceptual load. It requires continuous image monitoring, translating into a stronger response in the occipital brain regions.

Analysis of median tmax times for individual regions suggests that both protocols’ activation direction was identical. The earliest activity was recorded in the parietal cortex, followed by the prefrontal, motor, and visual cortices. This is consistent with other works [19,20]. This indicates a possible direction of activation: from integration of spatial information (parietal cortex) [21,22,23], through planning and executive control (prefrontal cortex) [24,25,26], and execution of the motor response (motor cortex) [27,28], to processing of visual information (visual cortex) [29]. In the passive task conditions, activation is more dispersed in time, and the most pronounced delays are observed in the visual cortex, which may indicate a lower level of processing stimuli and lesser involvement of attention.

A particularly interesting result is the occurrence of a significant, although weak Spearman correlation (R ≈ 0.2) between the activation amplitude and the time to reach its maximum; however, this was only the case in the motor and prefrontal cortex. This relationship was present only when analysing data from the passive task or all trials combined, and it did not occur in the interactive condition. It can be assumed that in functions with a low cognitive load and automatic character, the neuronal activation more reflects the “reactivity” of the participant’s nervous system: people with a larger HbO amplitude need more time to reach it. The absence of this relationship in the Nefroball task suggests that the processes controlling brain activity in dynamic conditions are modulated by the cognitive strategy and task requirements, not only by the physiological parameters of the response. The correlation is limited spatially exclusively to the motor and prefrontal cortex, confirming the functional importance of these structures for executive and control components. The lack of a similar effect in the parietal or visual cortex may be because spatial and sensory processing is strongly task-dependent and not subject to the same individual variability as motor control. Although the authors found no direct evidence that larger HbO amplitude is associated with longer latency in the work of other investigators, several studies suggest that larger HbO amplitudes are associated with greater cognitive effort. This leads to increased HbO amplitude in the prefrontal cortex, indicating greater cognitive effort and potentially a longer time to reach peak activation. Previous fNIRS-based studies have suggested similar interpretations [30,31,32].

The key result is the lack of significant differences in the magnitude of the maximum change in the concentration of oxygenated haemoglobin (HbO) in the motor, prefrontal, and parietal cortex. At the same time, the latency time was significantly shortened in the active condition. This relationship indicates that although the final intensity of activation may be similar in both tasks, the brain response appears faster in the interactive task. This mechanism can be explained by a higher level of attentional engagement and motivation when the participant performs actions that directly affect the events on the screen. The shortened latency time confirms the assumption that, in tasks requiring rapid eye–hand coordination (as in Nefroball), individual cortical areas—especially those responsible for planning and initiating movement—may be recruited more efficiently. This is consistent with the results of previous studies, in which interactive training protocols often lead to faster achievement of the threshold of neuronal activation than in purely passive conditions. This may be related to the participants’ higher level of attention and motivation, even though the maximum activation was similar in both conditions [33]. Previously published work suggests that active engagement in a task leads to faster and more dynamic activation of relevant brain areas, which may explain the shortened latency observed in the present study [33,34].

One limitation of this study is the focus on the left hemisphere only (due to technical limitations), which may not capture potential bilateral or right-dominant activations. Moreover, the homogeneity of the participant group (healthy young adults) may limit generalisability to clinical populations. Finally, as with all fNIRS studies, the signal reflects a combination of neuronal and vascular responses, and future studies should consider multimodal approaches (e.g., EEG-fNIRS) to disambiguate these effects. fNIRS measures haemodynamic changes that are secondary to neuronal activity, so it does not allow for a precise determination of the time of activation onset. Incorporating EEG could allow for the separation of sources of delays related to neuronal processing from those resulting from the vascular response.

Another limitation of the present study is the absence of behavioural data (e.g., hit rate, timing accuracy), which could have enriched the interpretation of haemodynamic responses. Future work will include synchronised behavioural performance logging to explore the relationship between task execution and cortical dynamics.

The participants were healthy young adults, which limits the possibility of extrapolating the results to clinical populations. However, it is planned to conduct a study in patients, such as those who have suffered a stroke, to assess the usefulness of the tmax index in evaluating the effectiveness of rehabilitation.

Additionally, deoxyhaemoglobin and total haemoglobin signals were not included in the current analysis. It should be noted that the statistical results were not corrected for multiple comparisons, which may increase the risk of a Type I error. However, the consistency of tmax effects across all regions strengthens the reliability of the observed pattern. All these limitations are planned to be taken into account in future studies.

The presented results seem to be important from a practical point of view, as they confirm that latency measurement using fNIRS can be a valuable complement to standard fNIRS signal analysis. Second, such studies can help optimise rehabilitation systems by providing information on how quickly specific areas of the cerebral cortex are activated during a task. This may be particularly important when designing therapeutic games, where an effective combination of motor and visual stimuli is crucial. In summary, the studies show that interactive tasks (such as NefroBall) significantly affect the dynamics of brain activation. Although the final level of response in most of the analysed areas may be similar to that in the passive task, the faster activation of neuronal processes and stronger stimulation of the visual cortex suggest that interaction with the virtual environment more intensively engages brain resources. In further research, it is worth extending the analysis to include a greater variety of tasks and participant groups to understand better the extent to which different forms of interaction translate into the effectiveness of activating specific cortical regions.

## Figures and Tables

**Table 1:** Results of the Shapiro–Wilk normality test for ΔoxyHb and tmax in each cortical region, separately for the interactive (A) and passive (P) protocols. W = Shapiro–Wilk statistic; *p* = significance level *p* < 0.05, indicating non-normal distribution.

The Analysed Area of the Cerebral Cortex	Protocol A				Protocol P			
	ΔoxyHb [mmol/L]		tmax [s]		ΔoxyHb [mmol/L]		tmax [s]	
	W	*p*	W	*p*	W	*p*	W	*p*
**Left hemisphere**	0.92	<0.001	0.90	<0.001	0.95	<0.001	0.94	<0.001
**Motor cortex**	0.92	<0.001	0.87	<0.001	0.96	0.003	0.90	<0.001
**Prefrontal cortex**	0.91	<0.001	0.88	<0.001	0.95	0.001	0.90	<0.001
**Parietal cortex**	0.92	<0.001	0.87	<0.001	0.91	<0.001	0.88	<0.001
**Visual cortex**	0.94	0.001	0.92	<0.001	0.93	<0.001	0.93	<0.001

**Table 2:** Statistical data for protocol A. ΔoxyHb [mmol/L] is the average amplitude of the increase in oxygenated oxyhaemoglobin, and tmax [s] is the latency time, i.e., the time from the beginning of the task to the HbO signal reaching its maximum value.

The Analysed Area of the Cerebral Cortex	ΔoxyHb [mmol/L]		tmax [s]	
	Median	25th/75th Percentile	Median	25th/75th Percentile
**Left hemisphere**	0.000289	0.000214/0.000423	5.250	4.250/7.500
**Motor cortex**	0.000421	0.000300/0.000564	5.500	4.750/8.250
**Prefrontal cortex**	0.000336	0.000236/0.000449	5.250	4.250/6.500
**Parietal cortex**	0.000287	0.000188/0.000416	4.875	3.500/6.750
**Visual cortex**	0.000336	0.000237/0.000497	5.625	3.750/9.875

**Table 3:** Statistical data for protocol P. ΔoxyHb [mmol/L] is the average amplitude of the increase in oxygenated oxyhaemoglobin, and tmax [s] is the latency time, i.e., the time from the beginning of the task to the HbO signal reaching its maximum value.

The Analysed Area of the Cerebral Cortex	ΔoxyHb [mmol/L]		tmax [s]	
	Median	25th/75th Percentile	Median	25th/75th Percentile
**Left hemisphere**	0.000252	0.000177/0.000382	6.250	4.750/9.500
**Motor cortex**	0.000379	0.000293/0.00055	6.500	5.250/10.250
**Prefrontal cortex**	0.000297	0.000208/0.000429	6.250	5.000/9.500
**Parietal cortex**	0.000238	0.000179/0.00037	5.750	4.125/10.500
**Visual cortex**	0.000305	0.000187/0.00039	8.875	4.625/11.125

**Table 4:** *p*-values from statistical comparisons of ΔoxyHb and tmax medians between the interactive (Nefroball—protocol A) and passive (observation—protocol P) conditions for all analysed areas.

The Analysed Area of the Cerebral Cortex	*p*-Coefficient/U Test Statistic	
	ΔoxyHb	tmax
**Left hemisphere**	0.090/4.041	0.016/3.763
**Motor cortex**	0.300/4.482	0.021/3.871
**Prefrontal cortex**	0.074/4.005	0.004/3.583
**Parietal cortex**	0.092/3.302	0.020/3.083
**Visual cortex**	0.031/2.849	0.016/2.769

**Table 5:** Information on the results of the correlation analysis between ΔoxyHb and tmax for all, protocol A, and protocol P results (the Spearman test). R—Spearman’s rank correlation coefficients. All values represent nonparametric rank correlations.

The Analysed Area of the Cerebral Cortex	All Results		Protocol A		Protocol P	
	R	*p*-Coefficient	R	*p*-Coefficient	R	*p*-Coefficient
**Left hemisphere**	0.14	0.186	0.12	0.261	0.12	0.261
**Motor cortex**	0.17	0.016	0.16	0.124	0.20	0.046
**Prefrontal cortex**	0.16	0.022	0.17	0.090	0.24	0.016
**Parietal cortex**	0.04	0.554	0.05	0.640	0.09	0.411
**Visual cortex**	0.04	0.571	0.21	0.054	−0.06	0.591

## Data Availability

Data supporting reported results can be found at https://doi.org/10.7910/DVN/HQIEDP (accessed on 27 June 2025).

## Abbreviations

The following abbreviations are used in this manuscript:

fNIRS	Functional near-infrared spectroscopy
tmax	The time from the beginning of the task to the HbO signal reaching its maximum value
HbO	Oxygenated reduced haemoglobin
HbR	Reduced haemoglobin
ΔoxyHb	The average amplitude of the increase in oxygenated oxyhaemoglobin
EEG	Electroencephalopathy

## References

[1] Ferrari M., Quaresima V. (2012). A Brief Review on the History of Human Functional Near-Infrared Spectroscopy (fNIRS) Development and Fields of Application. Neuroimage.

[2] Pinti P., Tachtsidis I., Hamilton A., Hirsch J., Aichelburg C., Gilbert S., Burgess P.W. (2020). The Present and Future Use of Functional Near-Infrared Spectroscopy (fNIRS) for Cognitive Neuroscience. Ann. N. Y. Acad. Sci..

[3] Lin P.-Y., Chen J.-J.J., Lin S.-I. (2013). The Cortical Control of Cycling Exercise in Stroke Patients: An fNIRS Study. Hum. Brain Mapp..

[4] Scholkmann F., Kleiser S., Metz A.J., Zimmermann R., Mata Pavia J., Wolf U., Wolf M. (2014). A Review on Continuous Wave Functional Near-Infrared Spectroscopy and Imaging Instrumentation and Methodology. Neuroimage.

[5] Leff D.R., Orihuela-Espina F., Elwell C.E., Athanasiou T., Delpy D.T., Darzi A.W., Yang G.Z. (2011). Assessment of the Cerebral Cortex during Motor Task Behaviours in Adults: A Systematic Review of Functional Near Infrared Spectroscopy (fNIRS) Studies. Neuroimage.

[6] Mihara M., Miyai I. (2016). Review of Functional Near-Infrared Spectroscopy in Neurorehabilitation. Neurophotonics.

[7] Koenraadt K.L.M., Roelofsen E.G.J., Duysens J., Keijsers N.L.W. (2014). Cortical Control of Normal Gait and Precision Stepping: An fNIRS Study. Neuroimage.

[8] Herff C., Heger D., Fortmann O., Hennrich J., Putze F., Schultz T. (2014). Mental Workload during N-Back Task—Quantified in the Prefrontal Cortex Using fNIRS. Front. Hum. Neurosci..

[9] Paus T. (2001). Primate Anterior Cingulate Cortex: Where Motor Control, Drive and Cognition Interface. Nat. Rev. Neurosci..

[10] Desmurget M., Sirigu A. (2009). A Parietal-Premotor Network for Movement Intention and Motor Awareness. Trends Cogn. Sci..

[11] Haggard P. (2008). Human Volition: Towards a Neuroscience of Will. Nat. Rev. Neurosci..

[12] Henschke J.U., Pakan J.M.P. (2023). Engaging Distributed Cortical and Cerebellar Networks through Motor Execution, Observation, and Imagery. Front. Syst. Neurosci..

[13] Borra E., Gerbella M., Rozzi S., Luppino G. (2017). The Macaque Lateral Grasping Network: A Neural Substrate for Generating Purposeful Hand Actions. Neurosci. Biobehav. Rev..

[14] Andersen R.A., Buneo C.A. (2002). Intentional Maps in Posterior Parietal Cortex. Annu. Rev. Neurosci..

[15] Gharbawie O.A., Stepniewska I., Kaas J.H. (2011). Cortical Connections of Functional Zones in Posterior Parietal Cortex and Frontal Cortex Motor Regions in New World Monkeys. Cereb. Cortex.

[16] Ito J., Joana C., Yamane Y., Fujita I., Tamura H., Maldonado P.E., Grün S. (2022). Latency Shortening with Enhanced Sparseness and Responsiveness in V1 during Active Visual Sensing. Sci. Rep..

[17] Schembri P., Pelc M., Ma J. (2019). Comparison between a Passive and Active Response Task and Their Effect on the Amplitude and Latency of the P300 Component for Visual Stimuli While Using Low Fidelity Equipment. Annu. Int. Conf. IEEE Eng. Med. Biol. Soc..

[18] Kavroulakis E., van Kemenade B.M., Arikan B.E., Kircher T., Straube B. (2024). The Effect of Self-generated versus Externally Generated Actions on Timing, Duration, and Amplitude of Blood Oxygen Level Dependent Response for Visual Feedback Processing. Hum. Brain Mapp..

[19] Miller B.T., D’Esposito M. (2012). Spatial and Temporal Dynamics of Cortical Networks Engaged in Memory Encoding and Retrieval. Front. Hum. Neurosci..

[20] Hinkley L.B.N., Nagarajan S.S., Dalal S.S., Guggisberg A.G., Disbrow E.A. (2011). Cortical Temporal Dynamics of Visually Guided Behavior. Cereb. Cortex.

[21] Andersen R.A. (1995). Encoding of Intention and Spatial Location in the Posterior Parietal Cortex. Cereb. Cortex.

[22] Gottlieb J., Snyder L.H. (2010). Spatial and Non-Spatial Functions of the Parietal Cortex. Curr. Opin. Neurobiol..

[23] Sack A.T. (2009). Parietal Cortex and Spatial Cognition. Behav. Brain Res..

[24] Funahashi S. (2017). Prefrontal Contribution to Decision-Making under Free-Choice Conditions. Front. Neurosci..

[25] Jones D.T., Graff-Radford J. (2021). Executive Dysfunction and the Prefrontal Cortex. Continuum.

[26] Friedman N.P., Robbins T.W. (2022). The Role of Prefrontal Cortex in Cognitive Control and Executive Function. Neuropsychopharmacology.

[27] Cheney P.D., Fetz E.E. (1980). Functional Classes of Primate Corticomotoneuronal Cells and Their Relation to Active Force. J. Neurophysiol..

[28] Evarts E.V. (1968). Relation of Pyramidal Tract Activity to Force Exerted during Voluntary Movement. J. Neurophysiol..

[29] Grill-Spector K., Malach R. (2004). The human visual cortex. Annu. Rev. Neurosci..

[30] Ayaz H., Shewokis P.A., Bunce S., Schultheis M., Onaral B. (2009). Assessment of Cognitive Neural Correlates for a Functional Near Infrared-Based Brain Computer Interface System.

[31] Dray G. (2013). Prefrontal Cortex Activity during Motor Tasks with Additional Mental Load Requiring Attentional Demand: A near-Infrared Spectroscopy Study. Neurosci. Res..

[32] Fishburn F.A., Norr M.E., Medvedev A.V., Vaidya C.J. (2014). Sensitivity of fNIRS to Cognitive State and Load. Front. Hum. Neurosci..

[33] Li W., Zhu G., Jiang Y., Miao C., Zhang G., Xu D. (2024). Cortical Response Characteristics of Passive, Active, and Resistance Movements: A Multi-Channel fNRIS Study. Front. Hum. Neurosci..

[34] Carius D., Kaminski E., Clauß M., Ragert P. (2025). Dynamic Alterations in Cortical Activation during Motor Adaptation in Table Tennis Using Whole-brain fNIRS. Sci. Rep..

